# School absenteeism as a predictor of functional gastrointestinal disorders in children

**DOI:** 10.3389/fped.2024.1503783

**Published:** 2024-12-12

**Authors:** Seth M. Tersteeg, Stephen M. Borowitz

**Affiliations:** Department of Pediatrics, University of Virginia, Charlottesville, VA, United States

**Keywords:** functional gastrointestinal disorders, school absenteeism, children, abdominal pain (MeSH), catastrophization, gastrointestinal complaints

## Abstract

**Introduction:**

Chronic abdominal complaints are common in school-aged children. Most affected children do not have underlying organic diseases but suffer from functional gastrointestinal disorders. While many children with chronic abdominal complaints experience school problems, no prospective studies have examined if school absenteeism is more common among children suffering from functional as opposed to organic gastrointestinal disorders. The purpose of this study was to determine if there is an association between school absenteeism and functional gastrointestinal disorders in children presenting to a pediatric gastroenterology clinic with chronic gastrointestinal complaints.

**Methods:**

Over a single year, families of school-aged children presenting to a pediatric gastroenterology clinic with gastrointestinal complaints were asked how many days of school their child had missed in the previous month due to their symptoms. At least six months after their visit, each child's final diagnosis was established and categorized as a functional disorder or an organic disease. Differences between children suffering from each diagnosis type were compared using unpaired *t*-tests.

**Results:**

Children with functional gastrointestinal disorders were more likely to experience significant school absenteeism than children with gastrointestinal diseases. Missing more than three days of school in the month prior to their visit had a negative predictive value of 82% for a gastrointestinal disease and being homebound from school during the month prior to their visit had a negative predictive value of 88% for a gastrointestinal disease. As compared to children with functional disorders, those with organic diseases were more likely to have missed three or fewer days of school in the previous month (sensitivity = 93%) and to have attended any school in the previous month (sensitivity = 99%).

**Discussion:**

Our data suggest children with functional gastrointestinal disorders are more likely to experience significance school absenteeism than children suffering from organic diseases. We suspect this may be due to higher perceived levels of pain and symptom catastrophizing caused by the duration and character of the diagnostic process, as well as biopsychosocial characteristics of these children.

## Introduction

Chronic abdominal pain and constipation account for 30%–38% of acute care visits to pediatricians (1). The overwhelming majority (>95%) of children who present to their primary care provider due to chronic gastrointestinal complaints do not have any identifiable organic cause for their symptoms and are suffering from functional gastrointestinal disorders (FGIDs) (2). The predominance of chronic gastrointestinal complaints being due to FGIDs persists amongst children referred to pediatric gastroenterologists, with more than 50% of children undergoing evaluation by a pediatric gastroenterologist meeting diagnostic criteria for at least one FGID, the majority of which are pain-related FGIDs (3–5).

While it is reassuring most children experiencing chronic gastrointestinal complaints are not suffering a serious underlying disease and rather are suffering from a FGID, their symptoms are real and can have a very negative effect on the child's quality of life (6). Patients with FGIDs who seek medical attention perceive their symptoms to be as severe as patients suffering from organic gastrointestinal disease (6, 7). Additionally, FGIDs remain diagnoses of exclusion, and affected patients often suffer from symptoms for extended periods of time before a definitive diagnosis is made (8). The long period of time without a clear explanation for symptoms and/or the family's discomfort with the diagnosis of a FGID may exacerbate the child's and family's fears as to the cause of the symptoms, thereby contributing to both family dysfunction and school absenteeism (3, 6, 9, 10). There is evidence that delayed diagnoses of some FGIDs are associated with reduced responsiveness to treatment, further underscoring the utility in additional diagnostic tools to aid in identifying these conditions earlier (11).

In children with some non-gastrointestinal disorders, major school absenteeism is associated with diagnoses that are functional rather than due to an organic disease and there is research examining how school absenteeism relates to symptom severity and symptom resolution in these patients (12, 13). However, in patients with chronic gastrointestinal symptoms, there do not appear to be any studies examining the relationship between school absenteeism and likelihood of their symptoms being functional rather than due to an organic disease nor the potential diagnostic utility of the extent of school absenteeism. The objective of this study was to determine if there is an association between school absenteeism and the diagnosis of FGIDs vs. organic diseases in children.

## Materials and methods

Between September 2016 and June 2017, the families of all children between five and 19 years of age enrolled in school between the 1st and 12th grade presenting to the Pediatric Gastroenterology Clinic at the University of Virginia with gastrointestinal complaints were asked how many days of school their child had missed over the previous month due to their gastrointestinal symptoms. Children were considered “homebound” if they had not attended a single day of school (20 absences) over the past four weeks. At least six months after their visit, chart review was performed to ascertain each child's final diagnosis. Diagnoses were categorized as either FGIDs or organic gastrointestinal diseases. The extent of the diagnostic workup performed prior to and after their consultation varied from patient-to-patient, but tests commonly performed included complete blood count, routine chemistries, serum lipase, erythrocyte sedimentation rate, c-reactive protein level, iron studies, tissue IgA transglutaminase antibody level, total serum IgA, fecal calprotectin and upper and lower endoscopy with biopsies. The child was considered to be suffering from a FGID when evaluation by a pediatric gastroenterologist did not identify an inflammatory, anatomic, infectious, or allergic condition that could account for the child's gastrointestinal symptoms. Functional diagnoses included functional abdominal pain, irritable bowel syndrome, non-ulcer dyspepsia, functional nausea, abdominal migraine, chronic constipation and/or encopresis, rumination disorder, functional vomiting, and functional dysphagia. Differences in school absenteeism between children suffering from functional disorders and those suffering from organic diseases were compared using unpaired *t*-tests.

## Results

**Figure 1:**
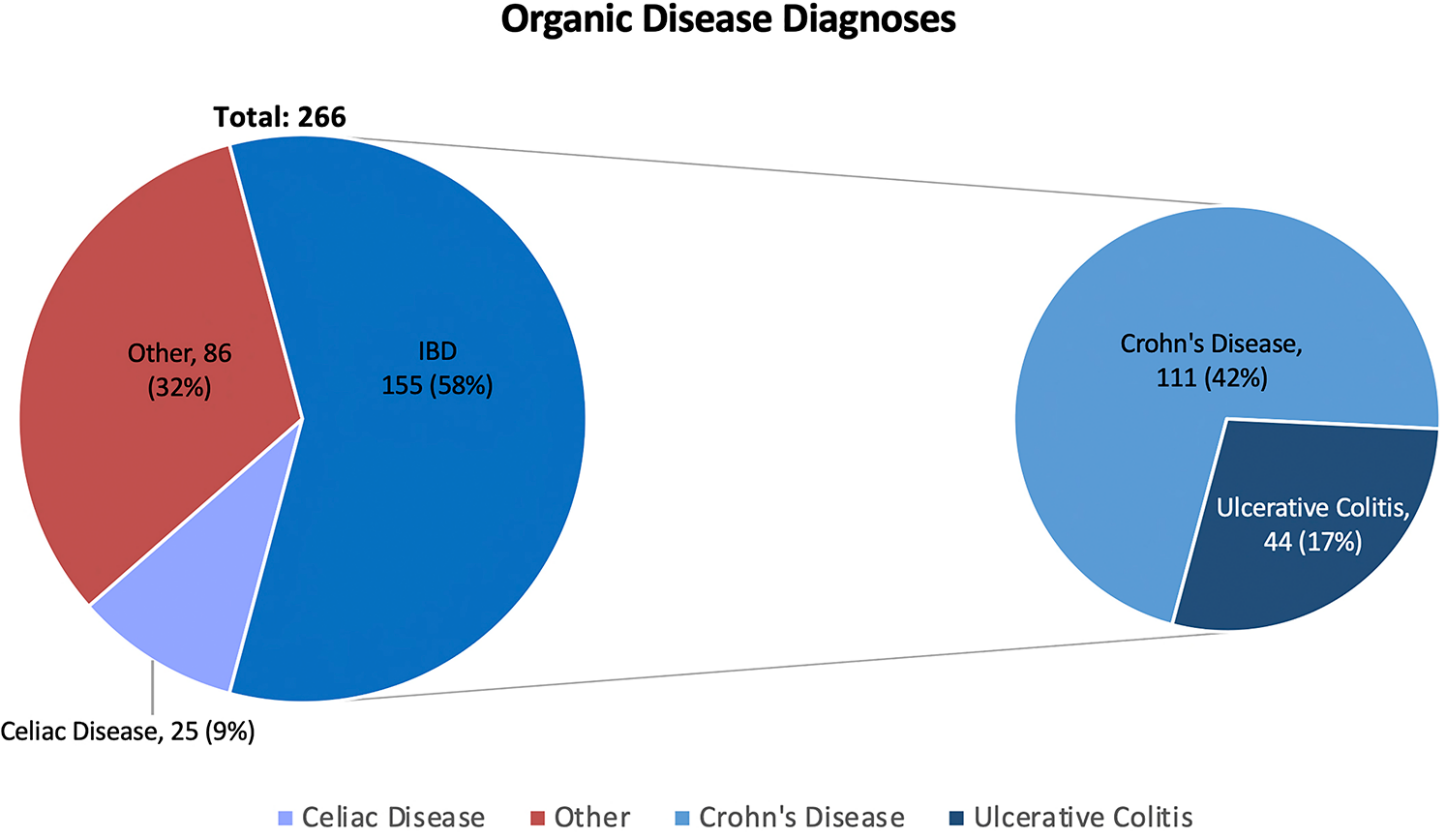
Percentage of the 266 organic disease patients receiving diagnoses of inflammatory bowel disease (IBD), celiac disease, or "other". IBD diagnosis are further divided into Crohn's disease and Ulcerative Colitis. Of the 50 children whose diagnoses were categorized as "Other", no more than 7 (<3% total) children received the same diagnosis.

**Figure 2:**
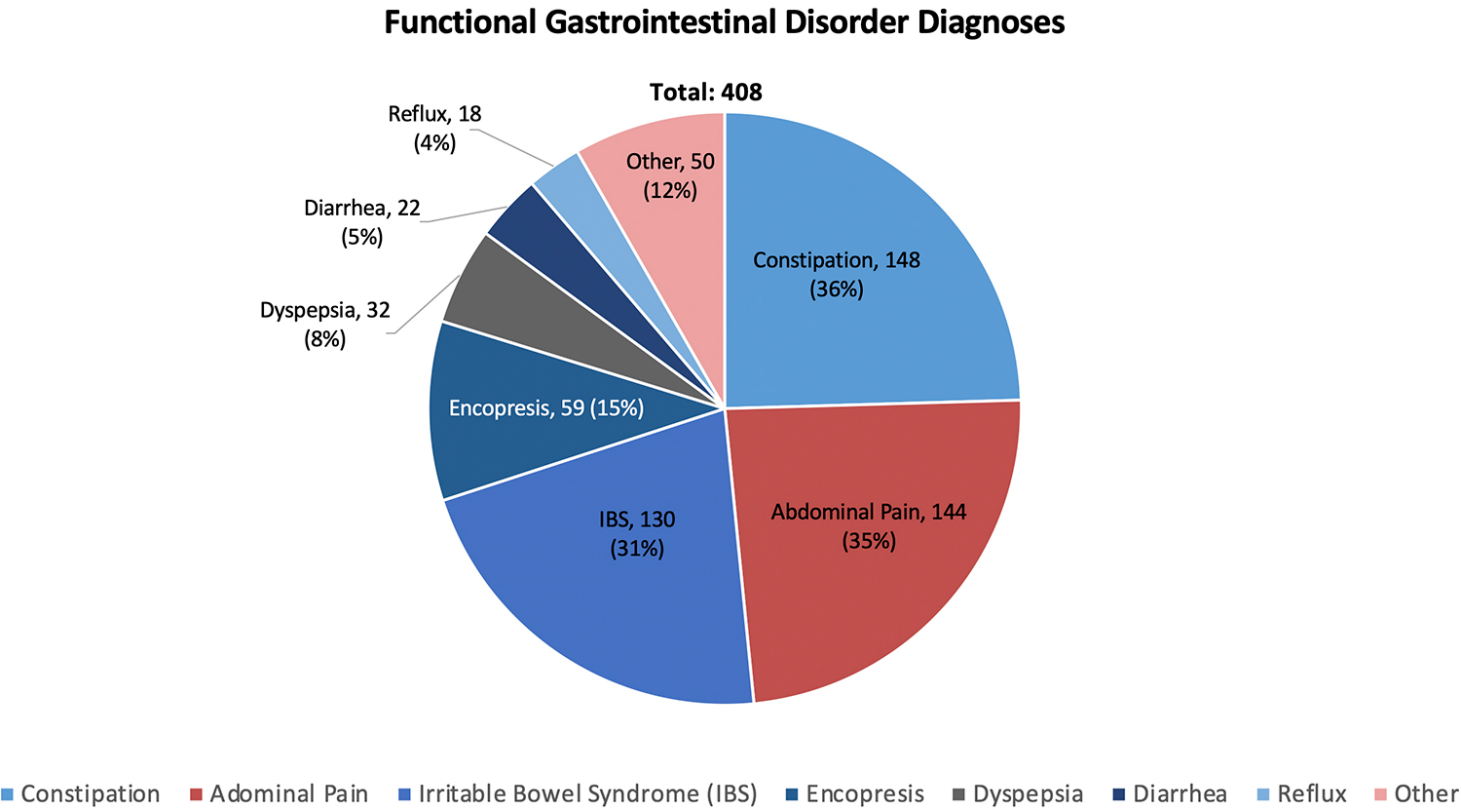
Percentage of the 408 FGID patients receiving specific diagnoses. Of the 50 children whose diagnoses were categorized as "Other", no more than 12 (<3% total) children received the same diagnosis. Importantly, a majority of these children (221, 54%) were diagnosed with multiple functional disorder.

During the study period, the authors completed 674 visits of school-aged children with chronic gastrointestinal complaints. 266 (39%) of these visits were due to organic diseases and 408 (61%) were due to FGIDs. Children suffering from functional disorders were more likely to be female (56% vs. 46%, *p* = 0.01) and were younger than those with organic diseases (11.2 ± 4.0 vs. 14.1 ± 3.8 years, *p* < 0.001) (Table 1). Of the 266 children ultimately diagnosed with organic diseases, 155 (58%) were diagnosed with inflammatory bowel disease (IBD), 25 (9%) were diagnosed with celiac disease, and 86 (32%) were diagnosed with a variety of other conditions including but not limited to gastroparesis, infectious/autoimmune hepatitis, H. pylori gastritis, and Juvenile Idiopathic Arthritis with gastrointestinal involvement. Of the 86 children identified as having one of these other conditions, no more than seven children had the same final diagnosis. These results are summarized in Figure 1. Of the 408 children ultimately diagnosed with FGIDs, the most common diagnoses were functional constipation (148, 36%), functional abdominal pain (144, 35%), and irritable bowel syndrome (IBS) (130, 31%). Additional diagnoses included functional encopresis (59, 15%), functional dyspepsia (32, 8%), diarrhea variant IBS (22, 5%), and gastroesophageal reflux disease (18, 4%). 50 (12%) children were diagnosed with miscellaneous other conditions including but not limited to migraine, functional vomiting, anxiety, rumination, autism spectrum disorder, dysphagia, and/or an eating disorder. No more than 12 of these 50 remaining children had the same final diagnosis. These results are summarized in Figure 2. Interestingly, 221 (54%) of 408 children diagnosed with a functional gastrointestinal disorder were diagnosed with more than one functional disorder. The degree of school absenteeism was not greater in the children diagnosed with multiple functional disorders than those diagnosed with a single functional disorder.

**Table 1:** Comparisons of age, gender, and average school absenteeism distributions for subjects in each diagnosis group.

	Children with Functional Disorders (*n* = 408)	Children with Organic Diseases (*n* = 266)	*p*-value
Age (years)			<0.001
Mean ± S.D.	11.2 ± 4.0	14.4 ± 3.7	
Median	11	15	
Range	5–19	5–19	
Female (%)	230 (56%)	123 (46%)	0.012
Male (%)	178 (44%)	143 (54%)	
Days of school missed in the past month (Mean ± SD)	2.8 ± 5.4	0.9 ± 3.3	<0.001

**Figure 3:**
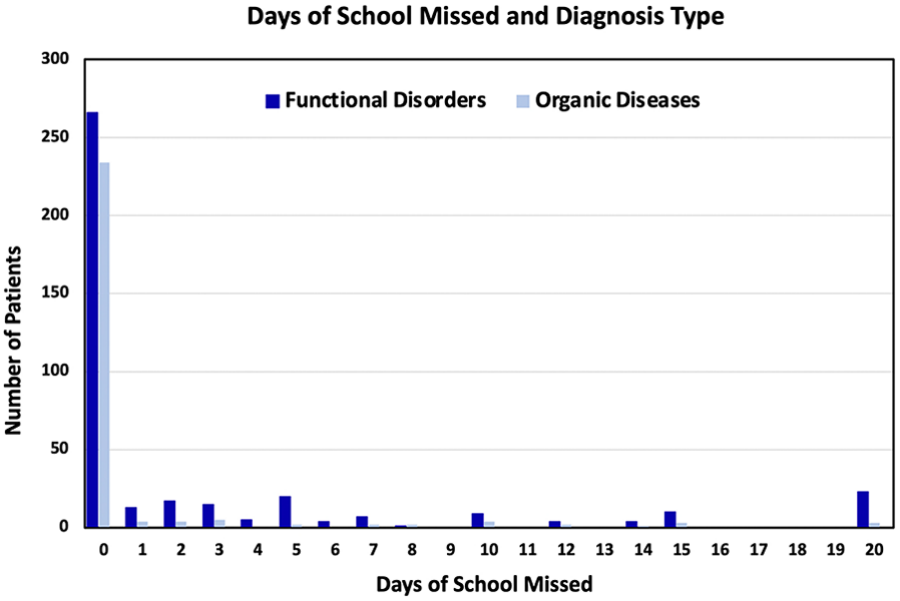
Bar graph of the number patients (*y*-axis) vs. the days of school they missed in the past month (*x*-axis), categorized by type of diagnoses they received (functional disorder or organic disease).

For 500 of the 674 visits (74%), the child had not missed any days of school in the previous month. 17 (3%) had missed one day of school, 21 (3%) missed two days of school, 25 (4%) had missed three days of school, and 106 (16%) had missed more than three days of school during the previous month. 26 (4%) were homebound, having not attended a single day of school over the previous month due to their gastrointestinal symptoms. These results are summarized in Table 2. As the number of school absences increased, the share of children diagnosed with FGIDs increased as compared to organic diseases. Of the 266 visits associated with organic diseases, 234 (88%) children had not missed any school over the previous month, four (1.5%) had missed one day of school, four (1.5%) had missed two days of school, five (1.9%) had missed three days of school, 19 (7.1%) had missed more than three days of school, and three (1.1%) were homebound. Of the 408 visits associated with a FGID, 266 (65.2%) had not missed any school over the previous month, 13 (3.2%) had missed one day of school, 17 (4.3%) had missed two days of school, 25 (6.1%) had missed three days of school, 87 (21.3%) had missed more than three days of school, and 23 (5.6%) were homebound. These results are summarized in Figure 3.

**Table 2:** Distribution of days of school missed in the previous month by number of visits (and percentage of total visits), with each visit representing 1 patient.

Days of school missed in the previous month	Number of visits (% of total visits)
0 days	500 (74%)
1 day	17 3%)
2 days	21 (3%)
3 days	25 (4%)
>3 days	106 (16%)
Homebound (0 days of school attended)	26 (4%)

**Figure 4:**
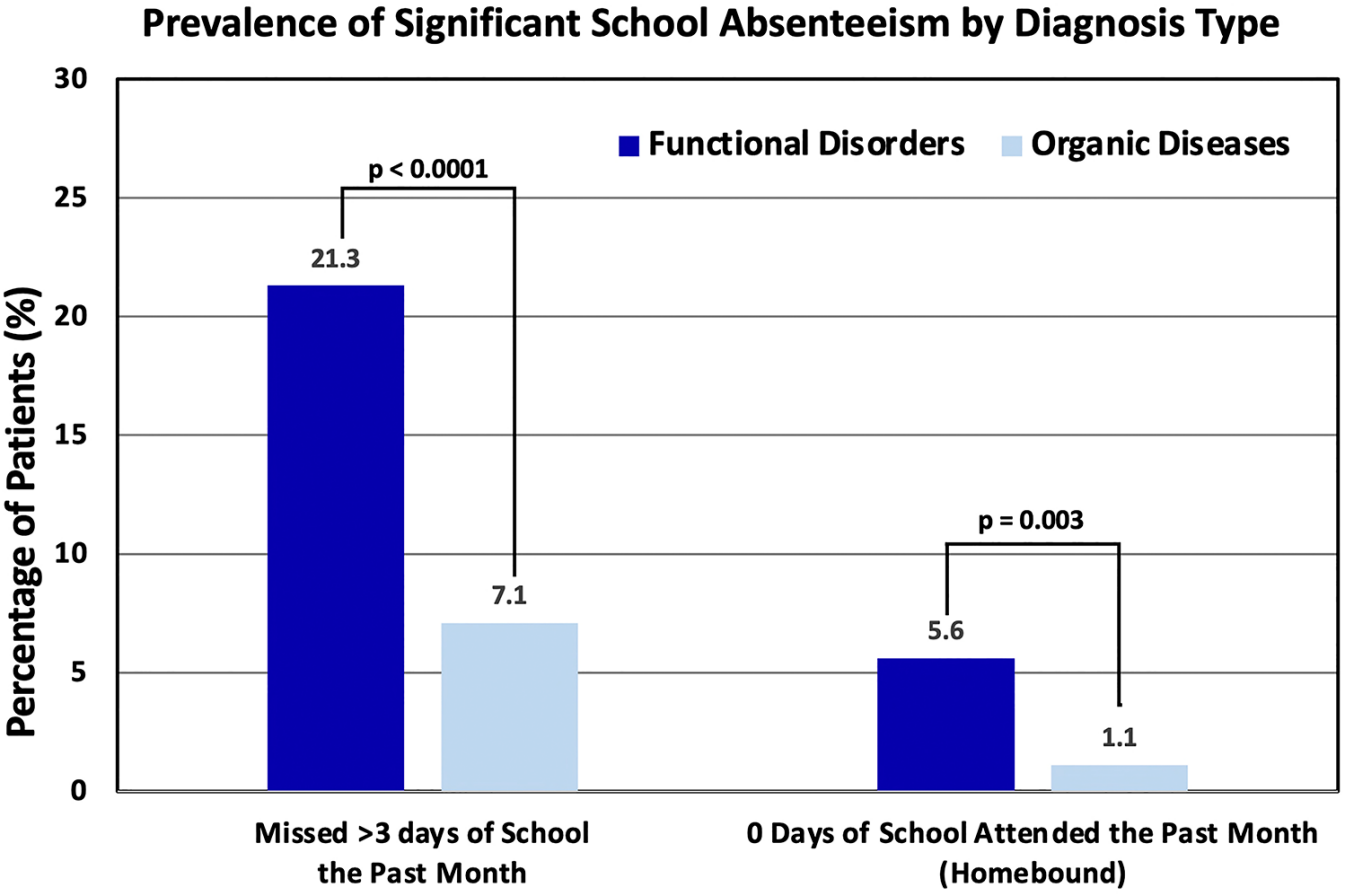
Percentage of patients categorized by diagnosis type (functional disorder or organic disease) vs. school attendance in the past month.

Children diagnosed with functional disorders were significantly more likely to have missed more than three days of school during the previous month than children with organic diseases (*p* < 0.001); 82% of children who had missed more than three days of school over the previous month were diagnosed with a FGID, as compared to 18% of children diagnosed with an organic gastrointestinal disease (Figure 4). Missing three or fewer days of school due to gastrointestinal symptoms had a sensitivity of 92.86% for organic disease (95% CI 89.07%–95.65%) and a negative predictive value (NPV) of 82.08% (95% CI 74.07%–88.01%). Of the 26 children who were homebound over the month prior to their visit, 23 were diagnosed with a functional disorder (*p* < 0.01) (Figure 4). Attending any school over the past month (in other words, not being homebound due to symptoms) had a sensitivity of 98.97% (95% CI 96.74%–99.77%) and NPV of 88.45% (95% CI 69.92%–96.20%) for organic disease. These results are summarized in Table 3.

**Table 3:** Sensitivity, specificity, positive predictive value, and negative predictive value for organic disease of (1) missing fewer than 3 days of school in the past month and (2) attending at least 1 day of school in the past month.

	Organic disease diagnosis	
	≤3 school absences (95% CI)	>0 days of school attendance (not homebound) (95% CI)
Sensitivity	92.86% (89.07–95.65%)	98.87% (96.74–99.77%)
Specificity	21.32% (17.45–25.62%)	5.64% (3.61–8.34%)
Positive predictive value (PPV)	43.49% (42.00–44.98%)	40.59% (39.94–41.24%)
Negative predictive value (NPV)	82.08% (74.07–88.01%)	88.46% (69.92–96.20%)
Disease prevalence	39.47%	

## Discussion

These data suggest major school absenteeism is significantly more common among children suffering from FGIDs than among those suffering from organic gastrointestinal diseases. Furthermore, as school absenteeism increases due to gastrointestinal symptoms, it appears increasingly likely the underlying cause of these symptoms is a FGID rather than an organic disease. In this study, missing more than three days of school over the previous month due to gastrointestinal symptoms had a NPV of 82% for the child suffering from an organic disease and being homebound over the previous month due to gastrointestinal symptoms had a NPV of 88% for the child suffering from an organic disease. While major school absenteeism did not demonstrate a NPV value high enough to exclude the possibility of the child suffering from an organic disease, it should be noted this cohort was encountered in a gastroenterology specialty clinic wherein the prevalence of organic diseases (266 of 674, 39% in this study) is much higher than what is observed in the primary care setting (1). Given this difference in pretest probability for organic disease, it is expected that the NPV of substantial school absenteeism would be higher in children presenting to their primary care provider with gastrointestinal complaints. The potential diagnostic utility of school absenteeism is further supported by the high sensitivity of school attendance; 93% of children with organic disease missed three or fewer days of school due to their symptoms and 99% of these children were able to attend some school during the previous month.

These data suggest that asking about school absenteeism as part of the evaluation of chronic gastrointestinal complaints may provide diagnostically useful information. Although significant school absenteeism did not have a particularly high positive predictive value (PPV) or specificity for identifying children with organic disease (Table 3), it would be more useful to have a test with very high sensitivity and NPV to help identify those children with chronic gastrointestinal symptoms who have a very low likelihood of suffering from an organic disease.

It is important to recognize that the majority of children in our study missed very little school as a result of their gastrointestinal symptoms. Nearly 75% of children with both FGIDs and organic gastrointestinal diseases missed no school over the previous month, and 80% of children missed three days of school or fewer over the previous month. For only 106 of the 674 visits (16%) did a child miss more than three days of school over the previous month and only 26 (4%) were homebound over the previous month (Table 2). Given our relatively small sample size, more data are needed to better understand the potential utility of school absenteeism to help distinguish between organic and functional causes of chronic gastrointestinal symptoms. However, further exploration of this association may provide some insight into the pathophysiology of FGIDs in some children.

The positive relationship between days of school missed due to gastrointestinal symptoms and the likelihood of a functional disorder raises an interesting question: why do children with FGIDs seem to miss more school than those with organic diseases? We hypothesize that the larger magnitude of school absences amongst children with FGIDs may serve as a proxy for perceived symptom severity and the likelihood of catastrophizing. Nearly two-thirds of children with FGIDs experience high levels of pain, and almost a quarter of these children exhibit high levels of pain catastrophizing (7). Pain catastrophizing is defined as a set of exaggerated, negative cognitive and emotional maladaptive schema brought to fruition by actual or anticipated painful stimulation. In other words, it is the tendency to magnify the threat or seriousness of pain sensations, often accompanied by an emphasis on pain-related fear and an inability to divert attention away from the pain or fear of pain (14). Pain catastrophizing (or catastrophizing in general) is associated with decreased quality of life, greater functional disability, increased risk for depression, and school absenteeism (9). In adults with FGIDs, catastrophizing is associated with high rates of work absenteeism and social impairment (7, 15). Our data suggest that some children and adolescents with FGIDs experience similar disruption in their lives as a result of their symptoms (3).

In some children with FGIDs, the length and extent of the diagnostic process coupled with the lack of a definitive “answer” for the cause of their symptoms and the seeming lack of an ability to cure their symptoms may contribute to the catastrophizing (10). Application of the Rome IV criteria, combined with elimination of “red flag” symptoms and signs is the standard means of diagnosing FGIDs in adults, however, the diagnostic accuracy of these criteria is less certain in the pediatric population. As compared to adults, the Rome IV criteria have decreased specificity and PPV in children and adolescents (8, 16). Because FGIDs remain diagnoses of exclusion, establishing with high certainty that a patient is suffering from a FGID rather than an organic disease may involve subjecting children to an extensive battery of tests and/or procedures without any positive results indicating the exact cause of their symptoms and how they can be addressed. Additionally, per the Rome IV criteria, most of the conditions categorized as FGIDs require patients to experience symptoms for at least two months, with some diagnoses requiring symptoms to persist for at least six months (8). The lengthy and involved diagnostic process is almost certainly emotionally taxing on patients and their families, as they may have symptoms for many months without an explanation or means of alleviating their symptoms. Furthermore, when enough diagnostic information has been collected for clinicians to confidently rule out organic disease in favor of a FGID, the result of this process not being a definitive explanation for their symptomatology may make patients feel that their concerns are being minimized or stigmatized. All of these factors, in combination with the understandable disappointment families may experience when their expectations of being cured are deemed unrealistic, can contribute to learned helplessness, hypervigilance to symptoms, and the catastrophizing observed amongst some patients suffering from FGIDs (6, 10). It would be interesting to know if the duration of time before a definitive diagnosis is established (and embraced) is greater among children with both FGIDs and organic diseases who miss substantial amounts of school than those who don't. Similarly, it would also be interesting to directly explore if length of symptomatology without receiving a diagnosis is related to likelihood to catastrophize.

Our study has a number of limitations. 53% of the children diagnosed with an organic gastrointestinal disease who missed more than three days of school over the previous month, as well as all three of the children who were homebound due to an organic disease, were diagnosed with inflammatory bowel disease. While this is a very small sample size (*n* = 3 for homebound children with organic disease), our data suggest children with organic diseases who miss large amounts of school due to their gastrointestinal symptoms are most likely to be suffering from inflammatory bowel disease. This raises another limitation of this study, which is the absence of systematically collected information about symptom severity (pain scale, stool frequency, stool caliber, presence of nausea/vomiting, etc.), initial symptom(s) at presentation (and whether patients were presenting with isolated or a combination of symptoms), or symptom duration prior to first presentation for their pediatric gastroenterology consultation and their relationship to school absenteeism. Although no particular group of FGIDs exhibited significantly greater absenteeism compared to the others in this study, including those ultimately diagnosed with multiple FGIDs, further studies may reveal stronger relationships between specific FGID diagnoses and school absenteeism. It would also be interesting to investigate whether this is a correlation between the duration of symptoms prior to initial consultation and the final diagnosis, however we did not collect those data.

It makes intuitive sense that children who miss more school have more severe/extensive disease, and there is some literature supporting the notion that both children with FGIDs (specifically, functional abdominal pain) and inflammatory bowel disease miss significantly more school than those with other chronic gastrointestinal complaints (1). It seems reasonable to suspect that patients suffering from FGIDs would follow this pattern as well, and those with more severe symptoms would experience more school absenteeism. We speculate major school absenteeism may have prognostic significance in for children suffering from FGIDs, as children who miss more school may have more severe symptoms and/or be more difficult to treat. If additional studies were to demonstrate a relationship between the extent of school absenteeism and the severity of symptoms and/or resistance to treatment, we posit substantial school absenteeism might aid in guiding both the diagnostic evaluation and therapeutic planning. Children exhibiting greater school absenteeism might warrant a more thorough and expedited diagnostic evaluation to quickly exclude organic disease and when the diagnostic evaluation proves negative, it might be appropriate to quickly move to intensive multimodal therapy to assure the best chance of success. Having said this, while major school absenteeism may point away from organic disease, the negative predictive value of school absenteeism is not sufficient to exclude an organic disease and children exhibiting severe or worrisome “red flag” symptoms or signs should certainly undergo diagnostic evaluation regardless of the extent of their school absenteeism.

Another limitation of this study is that we did not collect any data about other reasons for children missing school, such as bullying, difficulties at home, etc. It is very possible that the population of children suffering from FGIDs are disproportionately affected by other factors that could negatively influence school attendance. There is a well-established association between mental health conditions, such as anxiety and depression, and FGIDs in children (4, 17). This association persists into adulthood, as children with FGIDs are more likely to experience headaches/migraines, anxiety, and depression as adults than those without (4, 17, 18). A study examining school absenteeism in children with FGIDs found a predominance of children coming from low-income households, suggesting an inverse correlation between symptom severity and household income (5). This is significant, as it is well-established that children from low-income households are more likely to experience chronic school absenteeism (19). These potentially confounding factors are an additional limitation to our study, as students with FGIDs who missed more days of school may have had other reasons for their absenteeism, and the relationship between these other factors and gastrointestinal symptoms were not examined. Furthermore, these factors may obscure our ability to be certain that school absences that the children/families attributed strictly to gastrointestinal symptoms were solely due to those symptoms and not due to other social issues.

## Conclusion

Our data suggest children with chronic gastrointestinal complaints who experience significant school absenteeism are more likely to be suffering from a FGID than an organic disease. Missing more than three days of school over the previous month due to gastrointestinal symptoms was associated with a NPV of 82% for an organic disease. Similarly, children with organic diseases were very unlikely to have missed more than three days of school in the previous month due to their gastrointestinal symptoms (sensitivity = 93%). Children who remain homebound for an entire month of school due to gastrointestinal symptoms are even less likely to be suffering from an organic disease (sensitivity = 99%, NPV = 88%). Nevertheless, there are some children with gastrointestinal diseases, particularly inflammatory bowel disease, who miss a significant amount of school before their diagnosis is established. Given that the prevalence of organic disease amongst children presenting with chronic gastrointestinal complaints in the primary care setting is substantially lower than those referred for specialty evaluation, the presence of major school absenteeism (greater than three days in the past month) coupled with the absence of any “red flag” signs or symptoms makes the likelihood of serious organic disease very low.

## Data Availability

The raw data supporting the conclusions of this article will be made available by the authors, without undue reservation.
